# The Curvilinear Relationships Between Top Decision Maker Goal Orientations and Firm Ambidexterity: Moderating Effect of Role Experience

**DOI:** 10.3389/fpsyg.2021.621688

**Published:** 2021-04-15

**Authors:** Christopher Pryor, Susana C. Santos, Jiangpei Xie

**Affiliations:** ^1^Warrington College of Business, University of Florida, Gainesville, FL, United States; ^2^College of Business, Rowan University, Glassboro, NJ, United States; ^3^Business Research Unit, Instituto Universitário de Lisboa (ISCTE-IUL), Lisbon, Portugal; ^4^Department of Human Resource Management, Zhejiang Gongshang University, Xiasha University Town, Hangzhou, China

**Keywords:** ambidexterity, goal orientations, role experience, upper echelons theory, microfoundations, strategic entrepreneurship

## Abstract

Ambidextrous firms are those that can simultaneously manage exploitative and explorative innovation, which is why ambidexterity is key for firms that desire to pursue strategic entrepreneurship. Researchers have explored many of the reasons why some firms are more ambidextrous than others. However, little attention has been devoted to understanding how attributes of top decision makers can influence their firms' ambidexterity. By drawing on upper echelons theory and goal orientations research, we explain how firms' ambidexterity can be affected by top decision makers' motivations in achievement situations (i.e., goal orientations). Testing our hypotheses on a sample of 274 top decision makers of firms in the United States, we find that top decision makers' learning goal orientation – their desire to take risks and maximize learning–has an inverted U-shaped relationship with ambidexterity while top decision makers' performance prove goal orientation – their desire to demonstrate competence with existing skills – has a U-shaped relationship with ambidexterity. These effects are weaker for top decision makers who have greater role experience.

## Introduction

There has been increasing scholarly interest regarding firms' efforts to capture value from existing competencies while continuing to explore for new markets and means of creating value (Simsek et al., 2017; Withers et al., 2018). These efforts, which constitute strategic entrepreneurship, enable firms to simultaneously deepen their current competitive advantage (exploitation) while also seeking opportunities to introduce innovative products and services to new groups of customers (exploration) (Hitt et al., 2001; Ireland et al., 2003). Firms that successfully balance these activities are referred to as ambidextrous. Ambidexterity has been linked to superior innovative output (Raisch and Birkinshaw, 2008; Andriopoulos and Lewis, 2009; Wei et al., 2014) and performance (He and Wong, 2004; Lubatkin et al., 2006; Uotila et al., 2006).

A key component of firms' ambidexterity and ability to pursue strategic entrepreneurship is their leadership (Ireland et al., 2003; Gibson and Birkinshaw, 2004; Jansen et al., 2009; Kuratko and Audretsch, 2009). Top decision makers have the capacity to shape firms' behaviors, define their strategic objectives, and implement policies to enact their visions (Simsek et al., 2010; Heavey and Simsek, 2014). In particular, scholars have focused especially on understanding how top management teams and their behaviors may stimulate firms' ambidexterity (e.g., Lubatkin et al., 2006; Mihalache et al., 2014; Koryak et al., 2018). Additionally, research has explored the relationships between top decision makers' leadership characteristics and behaviors and ambidexterity, finding that leaders who can foster team cohesion and efficacy (Jansen et al., 2016), enact processes that balance creativity and performance (e.g., Andriopoulos and Lewis, 2009), practice transformational leadership (Jansen et al., 2008), and share leadership responsibilities across the top management team (Mihalache et al., 2014).

However, despite the importance attributed to top decision makers' cognitions on decision making and firm-level outcomes (Helfat and Peteraf, 2015; Liu et al., 2018), there has been much less focus on how top decision makers' cognitive attributes can influence firms' ambidexterity [with some notable exceptions (Wang et al., 2016; Mammassis and Kostopoulos, 2019; Kiss et al., 2020)]. Though ambidexterity scholars have made important progress in helping us understand how firms' top decision makers idiosyncratic attributes may influence firms' ambidexterity, the links between the cognitive characteristics of individual top decision makers and ambidexterity remains significantly weaker. This is a limitation of ambidexterity research because individual top decision makers wield significant influence in their firms and can have powerful effects on their firms' behaviors and performance (Wang et al., 2016; Neely et al., 2020). Therefore, exploring these links can provide a key explanation for why firms may exhibit different levels of ambidexterity.

**Figure 1:**
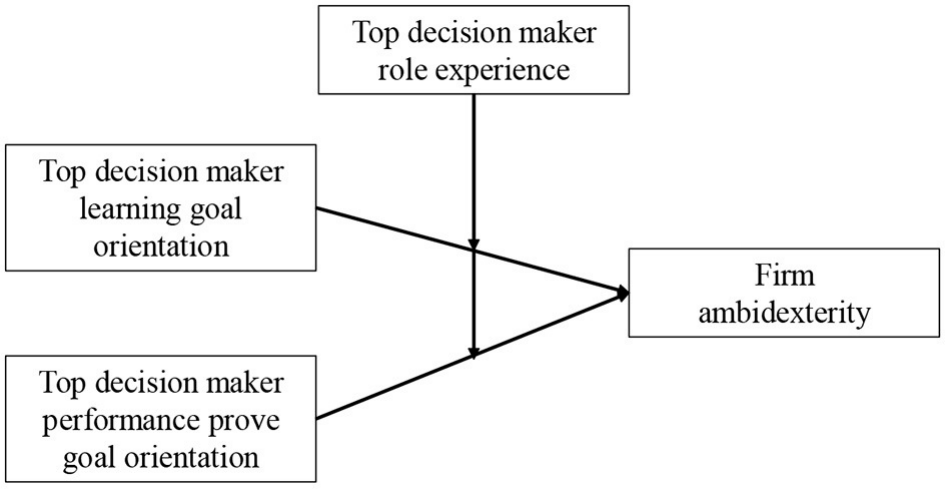
Top decision makers' goal orientation, role experience, and firm ambidexterity.

To address this limitation, we draw on upper echelons theory (Hambrick and Mason, 1984; Hambrick, 2007), which explains how the idiosyncratic characteristics of top decision makers influence their perception of and response to their firms' environment, and goal orientations research, which explains why people may exhibit different motivations when pursuing important tasks and how these motivations influence behavioral preferences (VandeWalle, 1997; DeShon and Gillespie, 2005; Hirst et al., 2009). As shown in Figure 1, we argue that top decision makers' learning goal orientation, defined as a preference to take risks and maximize learning (VandeWalle, 1997), is associated with improved firm ambidexterity up to a point, past which it is negatively related to firm ambidexterity (i.e., inverted U-shaped relationship). We also argue that top decision makers' performance goal orientation, defined as the preferences to demonstrate existing competencies to others (VandeWalle, 1997), is associated with reduced firm' ambidexterity up to a point, past which it is positively related to firm ambidexterity (i.e., U-shaped relationship). Furthermore, we contend that top decision makers' abilities and cognitive processes can improve with higher role experience (e.g., Graf-Vlachy et al., 2020), and we argue that greater role experience will flatten both the relationship between learning goal orientation and firm ambidexterity, and performance goal orientation and firm ambidexterity. We draw on a sample of 274 top decision makers of firms in the United States to test our hypotheses.

Our study contributes to research on strategic entrepreneurship and ambidexterity, goal orientations, and top decision makers. While early conceptualizations of strategic entrepreneurship tended to overlook the role of the top decision maker (e.g., Hitt et al., 2001), the top decision maker has played an increasingly prominent role in more recent conceptualizations (Withers et al., 2018). Nevertheless, empirical research concerning the role of the top decision maker in promoting strategic entrepreneurship – as well as ambidexterity – continues to be underdeveloped (Chen and Nadkarni, 2017). This paper strengthens the empirical record regarding the role of the top decision maker in promoting strategic entrepreneurship in their firms. Similarly, while ambidexterity research recognizes that top decision makers wield a powerful influence (Raisch and Birkinshaw, 2008), the role of the individual top decision maker has received significantly less attention than the top management team. While some studies have examined the effect of top decision maker characteristics on ambidexterity, such as social network extensiveness (Cao et al., 2010), and breadth of expertise (Wang et al., 2016). Our study is among the first to explore the effects of the idiosyncratic psychological differences among top decision makers on firms' ambidexterity.

Our study also contributes to goal orientations research. While research has generally supported positive relationships between learning goal orientation and performance (e.g., Payne et al., 2007; Lu et al., 2012; Pryor et al., 2019), our study suggests when it comes to learning goal orientation, it is possible to have “too much of a good thing” (Pierce and Aguinis, 2013). Learning goal orientation drives people's willingness to take risks, which can improve their performance. However, our findings suggest that learning goal orientation at higher levels may detract from people's (and firms') ability to enact desirable behaviors as these people either come to bear too much risk or focus on learning to the detriment of performing. Interestingly, this finding at the individual level is consistent with existing research on ambidexterity at the firm level, which emphasizes that balance between explorative and exploitative activities is preferable to too strong an emphasis on either one (e.g., March, 1991; Cao et al., 2010). Additionally, we propose that goal orientations' effects may be contingent upon top decision makers' experience, which has been associated with improved managerial competency and cognitive ability (Haynie et al., 2010; Graf-Vlachy et al., 2020). More experienced top decision makers, through greater awareness and cognitive processing, may be able to mitigate the effects that their learning and/or performance goal orientations can have on firm-level behaviors. Finally, upper echelons research, in particular, has been criticized for an excessive focus the effects of the immutable, observable characteristics of top decision makers, such as age, while focusing less on their personality or cognitive characteristics (Bromiley and Rau, 2016; Steinbach et al., 2019; Neely et al., 2020). Our study contributes to the recent and growing stream of research that focuses on top decision makers' cognitive attributes (e.g., Mannor et al., 2016; Pryor et al., 2019).

## Theory and Hypothesis

### Ambidexterity

To secure long-term survival, firms must be able to adapt to the changing conditions of their market and competitive environment (Tushman and O'Reilly, 2002; Sine et al., 2006). In organizational research, the notion that the pace of change facing firms today is rapidly increasing fostered the concept of strategic entrepreneurship: firms most likely to survive in changing conditions are those that can simultaneously exploit current opportunities while also searching for new opportunities (Hitt et al., 2001; Ireland et al., 2003; He and Wong, 2004; O'Reilly and Tushman, 2004). These objectives, exploitation and exploration, introduce variety through innovation and developing alternative strategies, while also reducing variety, through refining existing routines, perfecting existing product or service offerings, and improving existing customer relationships (Siren et al., 2012). Empirical evidence supports the relationship between the balance between exploration and exploitation – that is, ambidexterity – and firm performance (Junni et al., 2013).

Ambidexterity enables firms to more deftly respond to environmental dynamism and competitive intensity (Lavie et al., 2010). In environments rife with change, customer preferences can change more rapidly and competitors may introduce new innovations more quickly, which can render a firms' existing products and services outdated (Dess and Beard, 1984; Jansen et al., 2006). These dynamics create the necessity for firms to devote resources to exploration, and increasing competitive intensity highlights the value of exploitation. Firms facing aggressive competition may seek to deepen their relationships with existing customers and improve current product and service offerings (Jansen et al., 2006). While firms' environmental and competitive conditions may influence the necessity for them to practice ambidexterity, firms' response depends largely on their top decision makers' perception, interpretation, and decision making. Therefore, characteristics unique to individual top decision makers and how they make decisions may lead to differences among firms in terms of the ambidexterity they exhibit.

### Upper Echelons Theory

Firms' top decision makers – whether the C-suite officer, president, founder, or owner – wield enormous in the firms they lead, and upper echelons theory explains how top decision makers' unique characteristics influence firms' behaviors (Hambrick and Mason, 1984). In particular, the theory explains that top decision makers perceive, interpret, and respond to firms' environmental conditions by making strategic decisions, and it explains that top decision makers may vary in how they pass through this perceptual process due to individual idiosyncrasies, resulting in firm heterogeneity (Hambrick, 2007). Environmental conditions, such as competitive intensity, resource munificence, or pace of innovation, have been found to influence firms' ambidexterity (e.g., Uotila et al., 2006; Coombs et al., 2009), which highlights the necessity to understand the role top decision makers play in perceiving and responding to these conditions.

Top decision makers can affect firm-level behaviors via a variety of mechanisms. Top decision makers set the strategic vision of the firm and communicate this vision to other managers and employees, in turn shaping others' behavior to align with top decision makers' preferences (Pryor et al., 2019). Top decision makers also act as the cognitive nexus of the firm, bridging the people, processes, and information from across their firms and disseminating their interpretations and analyses back throughout the firm (Helfat and Peteraf, 2015). Top decision makers choose what kinds of resources to acquire and how to deploy them within the firm (Sirmon et al., 2007). In particular, top decision makers affect hiring decisions and they can provide incentives to managers and employees to enact the behaviors they desire (Finkelstein, 1992). Finally, top decision makers serve as an example to others in the firm, and managers and employees observe and replicate these behaviors (Marquis and Tilcsik, 2013).

### Goal Orientations

When top decision makers encounter situations in their firm that require the achievement of a task or performance, they may exhibit motivational differences that influence their behaviors. These motivational differences are referred to as goal orientations (DeShon and Gillespie, 2005), and in this study, we focus on two goal orientations: learning goal orientation and performance goal orientation (Button et al., 1996; VandeWalle, 1997). Learning goal orientation refers to people's preferences to maximize learning outcomes when engaged in an achievement task (VandeWalle, 1997). People who exhibit high learning goal orientation will be more willing to take risks, seek positive and negative feedback from others, experiment with new behaviors, ideas, or approaches, and respond positively to failure (Cron et al., 2005; Klein et al., 2006; Hirst et al., 2009). People who exhibit high performance goal orientation will also be motivated to seek feedback (albeit positive feedback that confirms their abilities) devote effort to demonstrating the superiority of their existing knowledge and abilities, and pursue high-performing outcomes (Porath and Bateman, 2006; Dietz et al., 2015). In addition, people high on learning goal orientation are self-referent because they compare their current abilities with their previous abilities; however, people high on performance goal orientation are other-referent because they compare abilities they demonstrated during an achievement task against others' exhibited abilities to assess their relative dominance (Dietz et al., 2015). Both goal orientations have been empirically linked to improved performance, as learning goal orientation facilitates learning, which can be translated into improved performance, whereas performance goal orientation facilitates the competitive drive to outperform others (Elliot et al., 2005; Yeo et al., 2009).

Learning goal orientation and performance goal orientation bear similarities to exploration and exploitation. Empirical research has also linked goal orientations to innovative output, via learning goal orientation's positive effect on learning and performance goal orientation's positive effect on performance and output (Gong et al., 2013). Moreover, learning goal orientation increases people's desire to improve their abilities while increasing their willingness to take risks and endure failure, and both attributes benefit innovative output (Janssen and Van Yperen, 2004). Learning and performance goal orientations have also been found to positively affect feedback-seeking and environmental scanning behaviors (Gong et al., 2017; Pryor et al., 2019) which can, in turn, boost firms' innovative output (e.g., de Stobbeleir et al., 2011). While goal orientations' effects have been consistently portrayed in research as linear, findings in ambidexterity theory suggest that too much risk taking in the pursuit of learning or too much focus on existing competencies can disrupt a balance that ultimately benefits firms' performance.

We argue that top decision makers' learning goal orientation will have an inverted U-shaped relationship with their firms' ambidexterity. Top decision makers with low learning goal orientation will be less likely to seek out and may even avoid challenging situations, less likely to take risks, or seek feedback to improve their abilities (St.-Jean et al., 2018). Low learning goal orientation can also be associated with a belief that a person's abilities are fixed and cannot be improved (Ames, 1992). For these reasons, we would expect that top decision makers with lower learning goal orientation would be relatively less likely to lead their firms to pursue innovation of any kind, explorative or exploitative, instead preferring to rely on existing products and services to satisfy customer demands. Moreover, research has found that integration between top decision makers and other managers and employees in the firm can foster ambidexterity (Lubatkin et al., 2006). Learning goal orientation is positively associated with better communication among team members, deeper understanding of team goals, and the mutual provision of feedback to improve individual members' and team performance (Gong et al., 2013). Therefore, we expect that firms with top decision maker who have low learning goal orientation will be less effective at fostering cross-firm integration, thus reducing ambidexterity.

However, as top decision makers' learning goal orientation increases, so does their propensity to take risks, seek feedback, learn from mistakes, and pursue innovation (Janssen and Van Yperen, 2004; Gong et al., 2013). People with higher learning goal orientation also tend to believe that their abilities are improvable with effort (Dweck and Leggett, 1988), suggesting that top decision makers higher on learning goal orientation may similarly believe the same is true of their firm: that existing products and services can be improved and that the firms' competencies can be developed via the exploration of wholly new resources, routines, and products and services. Top decision makers with higher learning goal orientation will also commit more time to seeking feedback and communicating with other members of their firms' management team (DeShon et al., 2004), increasing integration, which is an important foundation for firms' ambidexterity. Additionally, research has linked learning goal orientation specifically with the simultaneous effort to improve competencies regarding people's current abilities as well as efforts to improve competencies in new ability trajectories (Porath and Bateman, 2006; DeRue and Wellman, 2009). Consistent with this research, we would also expect that top decision makers with higher learning goal orientation may stimulate a greater degree of firm ambidexterity.

For top decision makers who exhibit the highest levels of learning goal orientation, however, we expect the relationship between learning goal orientation and ambidexterity to begin to decline. People with high learning goal orientation approach opportunities to try new activities, experiment, take risks, and learn from failure (Fang et al., 2019), and in excess, the costs of these preferences may begin to outweigh the benefits. Excessive information can produce abundant new information that the top decision maker, managers, and employees have difficulty processing (Hemp, 2009). An overload of information is difficult to process, and it can hamper the firm's ability to process existing tasks and respond to changing competitor actions (e.g., Bettis-Outland, 2012). As a consequence, firms may become less effective not just at perceiving environmental conditions but also at launching innovations. Information overload can also make it more difficult for the top decision maker, managers, and employees to exchange information among themselves, harming their ability to coordinate ambidexterity. This is consistent with empirical evidence that has found firms obtain diminishing returns by increasing their learning efforts (Bunderson and Sutcliffe, 2003). High learning goal orientation is associated with risk taking and learning from failures. While these behaviors produce positive learning and performance effects, excessive risk taking could lead to excessive failures, damaging the firm's innovative efforts (Chandrasekaran et al., 2012). Top decision makers with very high learning goal orientation may prefer exploration to exploitation, given that exploration satisfies their motivations to try new things and push the bounds of their existing competencies. Ambidexterity is a sensitive balance, and without deliberate efforts from top decision makers and managers, firms may begin to prefer one activity over the other (Tushman and O'Reilly, 1996). Therefore, we expect that when top decision makers exhibit very high learning goal orientation, they will be predisposed to pursuing exploration over exploitation, which can threaten firms' ambidexterity. Therefore, taken together, we hypothesize that top decision-makers' learning goal orientation will have an inverted U-shaped relationship with their firms' ambidexterity.

*Hypothesis 1: Top decision makers*' *learning goal orientation will have an inverted U-shaped relationship with firms' ambidexterity*.

We also argue that top decision makers' performance goal orientation will have a U-shaped relationship with their firms' ambidexterity. Top decision makers with low performance goal orientation will feel less urge to demonstrate the dominance of their existing competencies to others, although their preference to avoid risks or the appearance of incompetence will also be weaker (Dweck and Leggett, 1988). Performance goal orientation is also associated with the belief that one's competencies are fixed and that they cannot be improved with effort (Dweck and Leggett, 1988). While this belief may negatively impact the innovative output of a firm led by a top decision maker with higher performance goal orientation, it is less likely to be guiding the behaviors of firms led by a top decision maker with a lower performance goal orientation. For these reasons, top decision makers with low performance goal orientation may be expected to lead their firms to practice greater degrees of innovation, whether explorative or exploitative, relative to top decision makers who have higher performance goal orientation.

However, as top decision makers' performance goal orientation increases, so does their desire to acquire favorable evaluations from peers, other executives, and stakeholders, and so does their hesitancy to take risks because risks could lead to failure and negative evaluations (Dietz et al., 2015). One way these top decision makers attempt to ensure that they will acquire positive performance evaluations from others is to select tasks at which they believe they will succeed (Kohli et al., 1998; Tyson et al., 2009). As a consequence, we expect top decision makers with higher performance goal orientation to prefer to rely on their firms' existing products and services rather than pursue innovations, which could backfire, revealing top decision makers' low ability. Therefore, we expect that these firms are more likely to rely on exploitation rather than exploration – or simply avoid innovation altogether –reducing the ambidexterity they display. In addition, research has found that people with higher performance goal orientation may tend to hide knowledge from coworkers, due to knowledge's value and its ability to increase individual performance (Rhee and Choi, 2017). If top decision makers with higher performance goal orientation are similarly inclined, this could hamper their willingness to share information throughout the firm and its ability to practice ambidexterity.

For top decision makers who exhibit even higher levels of performance goal orientation, however, we expect that their firms' ambidexterity may begin to increase for several reasons. First, as compared to learning goal orientation, which fosters an internal-orientation (i.e., the top decision maker examines their own and their firms' improvement), performance goal orientation fosters an external orientation, in which the top decision maker and others attempt to derive evidence from the external environment concerning top decision makers' and firms' performance (Pryor et al., 2019). This external orientation may lead top decision makers to develop a greater awareness of their external environment and competitors' actions. When coupled with the top decision makers' motivation to succeed in competition (Dietz et al., 2015), these top decision makers may overcome their initial risk aversion to producing innovation and, instead, promote innovation in order to respond to competition. Second, performance goal orientation has been linked to people's proactive behaviors to differentiate themselves from competitors (Porath and Bateman, 2006), and it has been linked to greater planning behaviors (Mehta et al., 2009). When occurring in conjunction, proactive and planning behaviors may also enable top decision makers with higher performance goal orientation to overcome their resistance to innovate, foster cross-firm integration, and create the conditions in which ambidexterity can arise (e.g., Worthington et al., 2009). For these reasons, we hypothesize that top decision makers' performance goal orientation will exhibit a U-shaped relationship with their firms' ambidexterity.

*Hypothesis 2: Top decision makers' performance goal orientation will have a U-shaped relationship with firms' ambidexterity*.

### Moderating Effect of Top Decision Makers' Role Experience

We argue that the inverted U-shaped effect of learning goal orientation and the U-shaped effect of performance goal orientation will be flatter for top decision makers who have greater experience in their roles than for less-experienced top decision makers. As top decision makers acquire experience in their leadership positions, they acquire information, learn new and refine existing skills, and can developed enhanced cognitive skills (Ng and Feldman, 2010; North, 2019). In this section, we focus on two ways that top decision makers' experience in their roles may mitigate the effects that their goal orientations may have their firms' behaviors.

First, as top decision makers acquire experience, they become better strategic decision makers, which means they are better able to identify and exploit market opportunities and marshal resources to adapt to changing environmental conditions (Dragoni et al., 2011). Experienced top decision makers will have a better sense of who they can rely on inside their organizations to provide useful feedback (Reagans and McEvily, 2003). Because ambidexterity involves paradoxical, competing functions in the firm, which can pit various divisions, departments, or managers and employees against the others, the experienced top decision maker may be able to rely on their greater knowledge of the people and their interests in the firm to either avoid or overcome these challenges (Gupta et al., 2006). Experience also contributes to top decision makers' skillset. Through repetitive and extensive exposure to similar experiences and variations – handling the day-to-day challenges of leading an organization – top decision makers can become deft problem solvers (Dragoni et al., 2011). As a consequence, top decision makers become more confident in their skills, willing to take risks, and innovate (Alvesson and Spicer, 2012). Finally, experienced top decision makers have a more nuanced understanding of their surrounding environment, which means they may be able to understand which kinds of goals to pursue, which problems to focus on, and how best to create ambidexterity in response to the environment, despite its inherent challenges and contradictions (Mom et al., 2015).

The improved strategic decision making, which top decision makers acquire through experience, can flatten goal orientations' effects. For the top decision maker with high learning goal orientation, greater experience and decision making ability can offset the risks they may take concerning product development (Custodio et al., 2019). Experience as the top decision maker can also contribute to their ability to process information (Rodenbach and Brettel, 2012), which can offset the consequences of information overload that might occur for top decision makers with high learning goal orientation. Greater role experience can also flatten the relationship between top decision makers' performance goal orientation and firm ambidexterity. Performance goal orientation leads people to select tasks at which they know they will succeed, and because role experience increases top decision makers' strategic decision making abilities, the tasks selected by a top decision maker with high performance goal orientation may be more challenging – such as implementing or managing ambidexterity – than those with lower role experience. Relatedly, performance goal orientation is associated with an increased hesitancy to take risks. However, top decision makers with greater abilities and self-efficacy, which are obtained through experience, may be less affected by risk (Mangos and Steele, 2001), mitigating the downside of performance goal orientation.

Second, top decision makers may, through their experience, become aware of their motivations and understand how those motivations enable and hamper their firms' performance. Specifically, experienced top decision makers may develop metacognitive awareness, which is the “general level of awareness one has concerning their own cognitions” (Haynie et al., 2010: p. 221). People with greater metacognition have an ability to identify, in the midst of an achievement situation, how their cognitive patterns are influencing their performance, and they are able to consciously alternate their cognition to better suit the situation or task (Ford et al., 1998; Bruning and Campion, 2018). Research has linked metacognitive capacity with greater self-assessment and self-efficacy (Bell and Kozlowski, 2008), and importantly, research has linked metacognition to the detection and correction of errors (Keith and Frese, 2005). Finally, metacognition may be a key to helping top decision makers balance exploration and exploitation activities because it can provide them with a greater conscious awareness of how their personal behaviors connect to firm behaviors, how resources are allocated to exploration and exploitation, and how best to respond to environmental conditions (Schmidt and Ford, 2003).

We also expect that metacognition, which is acquired through experience, can flatten the effects goal orientations have on firm ambidexterity. Metacognition concerns people's ability to monitor and control their thinking and behaviors (Roebers, 2017). There are several types of metacognitive control: (1) attention control refers to people's metacognitive ability to target their attention in appropriate directions, (2) encoding control refers to people's metacognitive ability to break down a problem into its constituent pieces and focus their efforts on the more important pieces of the problem, (3) information processing control refers to people's metacognitive ability to consume or communicate information quickly and accurately, (4) motivation control refers to the metacognitive ability people have to stimulate themselves to action, such as by thinking or talking aloud about future rewards and consequences, (5) emotion control refers to the metacognitive ability people have to diminish negative motions and reassure themselves when under stress, and (6) environmental control refers to tactics people may use to marshal elements in the environment to promote improved performance, such as by eliminating distractions from a workspace or by deliberately befriending high-performing colleagues (e.g., Corno, 1986). When wielded by experienced top decision makers, these control tactics can help them become aware of consequences of their goal orientations and implement practices to offset their downsides. For example, a study has found that top decision makers who experience greater levels of anxiety will build a team of managers to buffer them from risk exposure (Mannor et al., 2016). An experienced top decision maker with moderate levels of performance goal orientation could, therefore, replicate this strategy so that their personal exposure or awareness of the risks related to their firm's ambidextrous innovative activities may be fielded by others. The top decision maker with high levels of learning goal orientation, aware that they may prefer to consume new information and take excessive risks, may force themselves, through motivation control, such as setting deadlines, to execute a particular plan rather than to continue to explore for new options. Taken together, we expect that experienced top decision makers may exhibit greater metacognition, which can mitigate the downsides related to their goal orientations, flattening their effects.

*Hypothesis 3: Top decision maker role experience will moderate the inverted U-shaped relationship between top decision maker learning goal orientation and firm ambidexterity such that the relationship will be flatter for top decision makers with more role experience*.

*Hypothesis 4: Top decision maker role experience will moderate the U-shaped relationship between top decision maker learning goal orientation and firm ambidexterity such that the relationship will be flatter for top decision makers with more role experience*.

## Methods

### Sample and Procedure

We tested our hypotheses using a sample of top decision makers obtained from the alumni foundation database of a public university in the southern United States, which includes contact information for all of the alumni and non-alumni donors of the university. The database listed the employers and job titles for each alumn and/or donor, and we selected participants that had job titles that indicated they were among the top decision makers (i.e., C-suite executive, owner, president, etc.) in their firm. Using this selection criteria, we generated a mailing list with 2,468 names. During data collection, we followed Podsakoff et al. (2003) recommendations to use temporal and methodological separation to reduce the potential that common method bias may influence our results. Therefore, we collected data via two rounds of surveys, with about two months separating the rounds. The first round of surveys contained items used as control variables and to measure top decision makers' goal orientations and role experience. The second round of surveys contained items used to measure firms' ambidexterity.

The first round of surveys was sent to 2,468 participants. There were 456 participant responses to Round 1 surveys, and, of these participants, 274 fully responded to Round 2. This represents an overall response rate of 11.1%, which is similar to response rates reported in other survey-based research of top decision makers (e.g., Boone and Hendricks, 2009; Pryor et al., 2019). Of the top decision makers who completed both survey rounds, their average age was 55.90 years old (SD = 10.87), and about 83% were men. About 55% had earned a bachelor's degree, 33% had earned a master's degree, 7% had obtained a post-graduate degree (the remaining 5% had either not attended college or had not obtained a bachelor's degree). The firms represented in the sample were, on average, 41.04 years old (SD = 30.01) and employed 1,287.47 people (SD = 18,175.26). The firms in the sample represented a number of industries, including agriculture, oil and gas production, retail and other consumer services, finance, and property management and development. We used *t*-tests to compare top decision maker age, firm size, and firm age, and we found no significant differences between those who responded to the first survey round only and those who responded to both survey rounds. We used chi-squared tests to compare top decision maker gender and education, as well as firm industry, and we found no significant differences between those who responded to the first survey round only and those who responded to both survey rounds.

### Measures

Table 1 includes all of the items used for measures in the study.

**Table 1:** Items used in surveys.

**Variable**	**Survey item**
**FIRST ROUND SURVEY**	
Top decision maker age	What is your age?
Top decision maker gender	What is your gender?
Top decision maker education	What is your highest level of education? Please circle one: some high school, high school diploma/GED, some college/associates degree, vocational/tech degree, bachelor's degree, post-bachelors (masters) degree, Ph.D.
Firm age	When was your firm founded (year)? (Reverse coded)
Firm size	How many individuals are currently employed by your firm?
Industry	In what industry does your firm compete?
Role experience	How many years have you served as a top decision maker in your firm?
**Learning goal orientation**	
Item 1	I am willing to lead challenging projects form which I can learn a lot.
Item 2	I prefer to work in situations that require a high level of ability and talent.
Item 3	I often look for opportunities to develop new skills and knowledge.
Item 4	Developing my work ability (i.e., in terms of making decisions, dealing with important stakeholders, analyzing various sources of information, etc.) is important enough to take risks.
Item 5	At work, I enjoy challenging and difficult tasks where I'll learn new skills.
**Performance goal orientation**	
Item 1	I prefer to lead projects where I can prove my ability to others (i.e., decision makers in your firm or other firms, friends, family, etc.).
Item 2	I try to figure out what it takes to prove my ability to others (i.e., decision makers in your firm or other firms, friends, family, etc.).
Item 3	I enjoy when others at work (or those who are close to me) are aware of how well I am doing.
Item 4	I'm concerned with showing that I can perform better than others (i.e., other decision makers in your firm or competing firms).
**Variable**	**Item**
**SECOND ROUND SURVEY**	
Ambidexterity	Exploration ^*^ Exploitation
**Exploration**	
Item 1	Our firm accepts demands that go beyond existing products and services.
Item 2	We regularly search for and approach new clients in new markets.
Item 3	We experiment with new products and services in our local market.
Item 4	Our firm regularly uses new distribution channels.
Item 5	We frequently utilize new opportunities in new markets.
Item 6	We invent new products and services.
Item 7	We commercialize products and services that are completely new to our firm.
**Exploitation**	
Item 1	Lowering costs of internal processes is an important objective.
Item 2	We improve the efficiency of the ways we provide products and services.
Item 3	Our firm expands services for existing clients.
Item 4	We increase economies of scale in existing markets.
Item 5	We introduced improved, but existing products and services for our local market.
Item 6	We regularly implement small adaptations to existing products and services.
Item 7	We frequently refine the ways we provide existing products and services.

#### Goal Orientations

We measured learning goal orientation and performance goal orientation with items developed by VandeWalle (1997). For learning goal orientation, five items were used, including: “I am willing to lead challenging projects from which I can learn a lot” and “Developing my work ability (i.e., in terms of making decisions, dealing with important stakeholders, analyzing various sources of information, etc.) is important enough to take risks.” For performance goal orientation, four items were used, including: “I prefer to lead projects in which I can prove my ability to others” and “I'm concerned with showing that I can perform better than others (i.e., other decision makers in your firm or competing firms).” Participants responded to each item by using a 7-point Likert-type scale, which ranged from 1 = strongly disagree to 7 = strongly agree. The Cronbach alpha for the five items used to measure learning goal orientation was 0.77, and the Cronbach alpha for the four items used to measure performance goal orientation was 0.76.

#### Experience in Top Decision Maker Role

Participants' experience as a top decision maker in their firm was measured by their response to the question, “How many years have you served as a top decision maker in your firm?”

#### Ambidexterity

To measure firms' ambidexterity, we began by collecting participants' responses regarding their firms' exploratory innovation and exploitative innovation. Drawing from Jansen et al. (2006), we adapted seven items to measure exploratory innovation and seven items to measure exploitative innovation. Items measuring exploratory innovation included, “Our firm accepts demands that go beyond existing products and services” and “We invent new products and services.” Items measuring exploitative innovation included, “Lowering costs of internal processes is an important objective” and “We increase economies of scales in existing markets.” Participants responded to each item by using a 7-point Likert-type scale, which ranged from 1 = strongly disagree to 7 = strongly agree. The Cronbach alpha for exploratory innovation was 0.85, and the Cronbach alpha for exploitative innovation was 0.85. Participants' item responses were averaged to produce exploration and exploitation scores. Following prior research (He and Wong, 2004), we multiplied these scores to produce an ambidexterity score.

#### Controls

We controlled for top decision makers' age, gender, and education level because these characteristics may influence how they enact their role in the firm and influence firm-level outcomes (e.g., Wang et al., 2016). We also control for firm age (i.e., reverse coded from participants' response to the question: in what year was your firm founded?), size (i.e., coded from participants' response to the question: how many individuals are currently employed by your firm?), and industry (three dichotomous industry variables: agriculture and energy production, 1 = yes, 0 = no; business-to-business services, 1 = yes, 0 = no; consumer-oriented services, 1 = yes, 0 = no). Firm age was scaled down by 100 and firm size was scaled down by 1,000 so that their coefficients and standard errors would be consistent with other variables in the model. These controls were included because larger, older firms may have more resources with which to develop more effective strategies, such as those related to innovation, and because industries may vary in innovative activity (e.g., Dess et al., 1990; Xue et al., 2012).

## Results

We used Stata 14 to conduct a CFA for variables that were measured with participants' response to scaled items (learning goal orientation, performance goal orientation, explorative innovation, and exploitative innovation). As shown in Table 2, the four-factor model showed acceptable fit (Tucker-Lewis index = 0.94, confirmatory factor index = 0.94, standardized root mean residual = 0.06). All item loadings were significant, indicating convergent validity. We compared the four-factor model against alternative models and found the four-factor model to have significantly better fit, providing evidence for discriminant validity. A correlation matrix with all the variables' means and standard deviations is included in Table 3.

**Table 2:** Results of confirmatory factor analysis.

	**χ^2^**	***df***	**TLI**	**CFI**	**SRMR**
Four factor model	377.79	224	0.94	0.94	0.06
Three factor model (PGO-LGO, Exploration, Exploitation)	818.81	227	0.67	0.70	0.10
Three factor model (PGO-Exploration, LGO, Exploitation)	1094.04	227	0.52	0.57	0.18
Three factor model (PGO-Exploitation, LGO, Exploration)	794.51	227	0.68	0.72	0.09
Three factor model (PGO, LGO-Exploration, Exploitation)	815.60	227	0.67	0.70	0.10
Three factor model (PGO, LGO-Exploitation, Exploration)	815.19	227	0.67	0.71	0.10
Three factor model (PGO, LGO, Exploration-Exploitation)	545.59	227	0.82	0.84	0.07
Two factor model (PGO-LGO, Exploration-Exploitation)	838.60	229	0.66	0.69	0.10
One factor model	1155.46	230	0.49	0.54	0.12

*TLI, Tucker-Lewis index; CFI, comparative fit index; SRMR, standardized root mean square residual; PGO, Performance goal orientation; LGO, Learning goal orientation. N = 274*.

Table 3Means, standard deviations, and correlations.

**Table:** 

**Variable**	**Mean**	**Std. Dev**.	**1**	**2**	**3**	**4**
1. Top decision maker age	55.90	10.87				
2. Top decision maker gender	0.17	0.38	−0.07			
3. Top decision maker education	5.37	0.86	0.12^*^	0.00		
4. Firm age	41.04	30.01	0.10	−0.03	0.00	
5. Firm size	1287.47	18175.26	−0.08	−0.02	0.04	0.35^**^
6. Industry: Agriculture and energy production	0.05	0.23	0.00	−0.02	−0.05	0.01
7. Industry: Business-to-business services	0.49	0.50	0.01	−0.12^*^	−0.03	−0.06
8. Industry: Consumer-oriented services	0.45	0.50	−0.01	0.13	0.05	0.06
9. Role experience	16.60	10.52	0.52^**^	−0.10	−0.01	0.12^*^
10. Learning goal orientation	5.90	0.72	−0.05	0.02	0.04	−0.27^**^
11. Performance goal orientation	3.96	1.27	0.08	0.08	−0.02	−0.14^*^
12. Firm ambidexterity	25.28	8.56	0.15^*^	0.07	−0.01	0.03

**Table:** 

**Variable**	**5**	**6**	**7**	**8**	**9**	**10**	**11**
6. Industry: Agriculture and energy production	−0.02						
7. Industry: Business-to-business services	−0.05	−0.24^**^					
8. Industry: Consumer-oriented services	0.06	−0.22^**^	−0.90^**^				
9. Role experience	−0.10	0.21^**^	−0.04	−0.05			
10. Learning goal orientation	−0.41^**^	0.02	0.01	−0.02	−0.02		
11. Performance goal orientation	−0.12^*^	0.01	−0.02	0.01	0.00	0.15^*^	
12. Firm ambidexterity	0.07	−0.02	0.02	−0.02	0.12^*^	0.15^*^	−0.03

*N = 274*;

* *p < 0.05*,

** *p < 0.01*.

**Figure 2:**
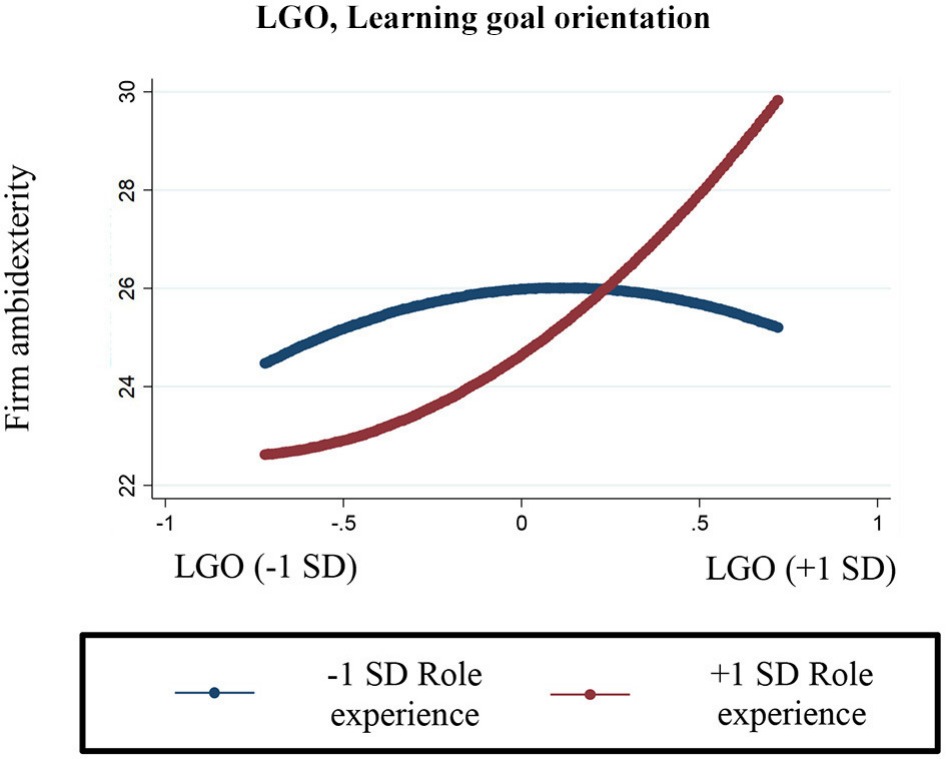
Interaction of top decision makers' learning goal orientation role experience on firm ambidexterity.

We used hierarchical regression to test our hypotheses. Interaction terms were centered before including in the analyses. Due to the presence of non-normal and heteroskedastic residuals, robust standard errors were used. Regression results are presented in Table 4. Model one includes the control variable regressed onto ambidexterity. Model two includes the hypothesized effects of top decision makers' goal orientations. Hypothesis 1 predicted that an inverted U-shaped relationship would exist between top decision makers' learning goal orientation and their firms' ambidexterity. That hypothesis was not supported (*B* = 0.51, *p* > 0.05). Hypothesis 2 predicted that a U-shaped relationship would exist between top decision makers' performance goal orientation and their firms' ambidexterity. That hypothesis was not supported (*B* = 0.37, *p* > 0.05). Hypothesis 3 predicted that the inverted U-shaped relationship between top decision makers' learning goal orientation and firm ambidexterity would be flatter for top decision makers with greater experience in their roles. Because we are using linear regression models, the moderation of U-shaped effects can be tested by assessing the significance of the coefficient of the interaction between the squared goal orientation term and the moderator (Haans et al., 2016). Therefore, we find support for this hypothesis because the coefficient reported in Model 3 for the interaction term between the squared learning goal orientation and top decision makers' role experience variables is significant and positive (*B* = 0.25, *p* < 0.01). This interaction is plotted in Figure 2, showing the effect of top decision makers' learning goal orientation (ranging from – 1 to +1 standard deviation[SD]) on firms' ambidexterity, given different levels of top decision makers' role experience (plotted at −1 and +1 SD, which corresponds to about 6 vs. 27 years of role experience, respectively). These effects are more precisely represented in Table 5, where we have included the marginal effects of top decision makers' goal orientations on firms' ambidexterity at −1 and +1 SD. For top decision makers with about 6 years of role experience, the relationship between learning goal orientation and ambidexterity begins with a positive slope (*B* = 3.68, *p* < 0.10) and ends on a weak downward slope (*B* = −2.67, *p* = n.s.). However, for top decision makers with about 27 years of role experience, the relationship between learning goal experience begins weakly positive (*B* = 0.61, *p* = n.s.) and ends on a strong upward slope (*B* = 9.40, *p* < 0.001). Although the curve is flatter for experienced top decision makers, which supports our hypothesis, the result does not suggest that the learning goal orientation of experienced top decision makers does not affect firms' ambidexterity. Indeed, what Figure 2 and Table 5 indicate is that experienced top decision makers appear to be able to extract greater benefits from their learning goal orientation, perhaps because they are able to control the downside consequences related to excessive risk taking.

**Table 4:** Hierarchical regression analysis, predicting firm ambidexterity.

	**Model 1**		**Model 2**		**Model 3**	
	***B***	**Robust Std. Err**	***B***	**Robust Std. Err**	***B***	**Robust Std. Err**
Constant	18.62	32.79	26.45	33.27	16.60	34.51
Top decision maker age	0.13^*^	0.06	0.14^*^	0.06	0.14^*^	0.06
Top decision maker gender	2.23	1.50	2.48	1.52	2.16	1.52
Top decision maker education	−0.47	0.64	−0.56	0.64	−0.54	0.65
Firm age	0.03	1.63	0.44	1.66	−0.06	1.72
Firm size	0.09^***^	0.02	0.04	0.05	0.30^*^	0.12
Industry: Business-to-business services	1.53	2.56	1.17	2.65	0.94	2.56
Industry: Consumer-oriented services	0.89	2.62	0.36	2.70	0.00	2.59
Role experience	0.06	0.06	0.06	0.06	0.04	0.08
Learning goal orientation	2.90^***^	0.77	2.75^**^	0.87	2.71^**^	0.81
Performance goal orientation	−0.42	0.42	−0.33	0.41	−0.30	0.40
Learning goal orientation squared (H1)			0.51	0.76	0.37	0.72
Performance goal orientation squared (H2)			0.37	0.29	0.40	0.27
Learning goal orientation ^*^ Role experience					0.21^**^	0.08
Learning goal orientation squared ^*^ Role experience (H3)					0.25^**^	0.08
Performance goal orientation ^*^ Role experience					0.00	0.04
Performance goal orientation squared ^*^ Role experience (H4)					−0.06^*^	0.03
F	28.88^***^		22.13^***^		15.92^***^	
R-squared	0.09		0.10		0.15	

*N = 274*;

* *p < 0.05*;

** *p < 0.01*;

*** *p < 0.001*.

**Table 5:** Marginal effects of top decision makers' goal orientations, moderated by role experience.

**Learning goal orientation**	**Low role experience (−1SD)**		**High role experience (+1SD)**	
**LGO score**	***B***	***SE***	***B***	***SE***
−1 SD	3.68‘	2.17	0.61	1.84
−0.5 SD	2.10	1.46	2.80^*^	1.26
Mean	0.51	1.23	5.00^***^	1.19
+0.5 SD	−1.08	1.67	7.20^***^	1.69
+1 SD	−2.67	2.45	9.40^***^	2.43
**Performance goal orientation**	**Low role experience (−1SD)**		**High role experience (+1SD)**	
**PGO score**	***B***	***SE***	***B***	***SE***
−1 SD	−2.93^**^	1.07	0.38	1.17
−0.5 SD	−1.63^*^	0.71	0.05	0.8
Mean	−0.31	0.59	−0.29	0.61
+0.5 SD	1.02	0.83	−0.62	0.77
+1 SD	2.32‘	1.23	−0.96	1.13

*N = 274; ‘p < 0.10*,

* *p < 0.05*,

** *p < 0.01*,

*** *p < 0.001*.

**Figure 3:**
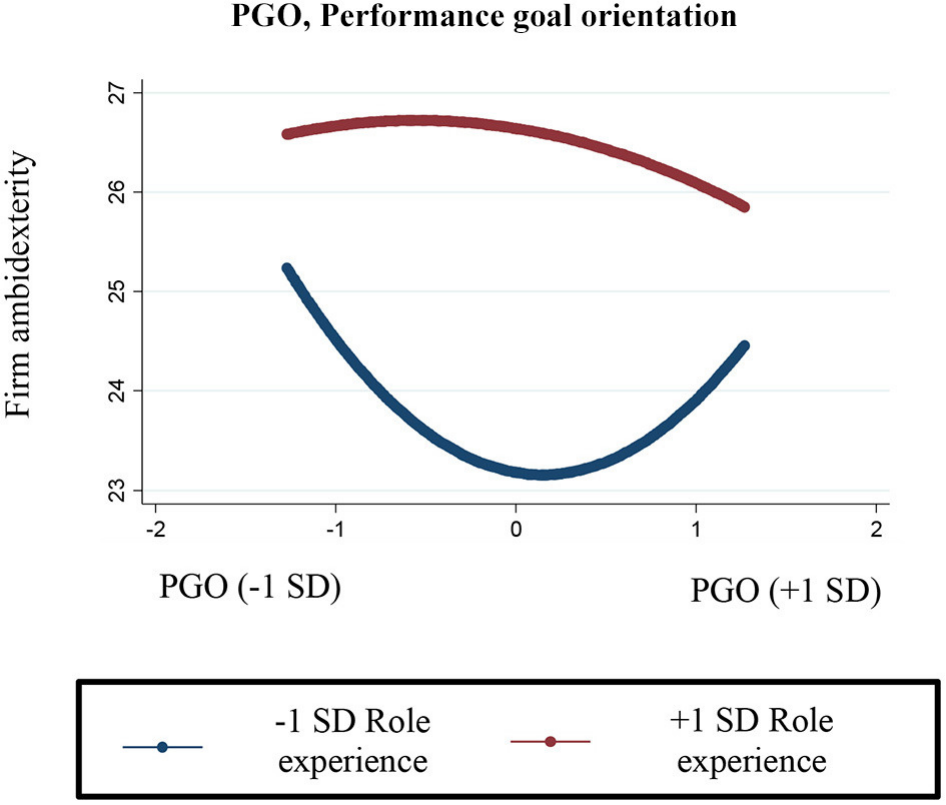
Interaction of top decision makers' performance goal orientation and role experience on firm ambidexterity.

Hypothesis 4 predicted that the U-shaped relationship between top decision makers' performance goal orientation and firms' ambidexterity would be flatter for top executives with greater role experience. This hypothesis was also supported (*B* = −0.06, *p* < 0.05). This interaction is plotted in Figure 3, which shows the relationship between top decision makers' performance goal orientation (ranging from −1 to +1 SD) on firms' ambidexterity, given different levels of top decision makers' role experience (plotted at −1 and +1 SD). The marginal effects of the relationships are also included in Table 5. For top decision makers with about 6 years of role experience, the relationship between performance goal orientation and ambidexterity begins negatively (*B* = −2.93, *p* < 0.01) and ends positively (*B* = 2.32, *p* < 0.10). However, for top decision makers with about 27 years of role experience, the marginal effects across the whole relationship are weaker when compared to the relationship for less-experienced top executives, suggesting that increased role experience flattens the effect of performance goal orientation so that it has no effect on firms' ambidexterity.

## Discussion

Ambidexterity – a firm's capacity to simultaneously undertake exploration and exploitation–is an important component of strategic entrepreneurship, which emphasizes deepening existing strengths while also exploring for new opportunities (Hitt et al., 2001; Ireland et al., 2003). Researchers have argued that “ambidextrous organizations need ambidextrous senior teams and leaders” (O'Reilly and Tushman, 2004: p. 81). While there has been significant exploration concerning the role of top management teams and firm ambidexterity, there has been little research linking the attributes of the individual top decision makers in firms to ambidexterity, with a few important exceptions (e.g., Laureiro-Martinez et al., 2015; Mammassis and Kostopoulos, 2019). Therefore, our study contributes to ambidexterity research by describing and testing the relationships between top decision makers' learning goal orientation and performance goal orientation and firm ambidexterity, across top decision makers of varying role experience.

We find that, for less experienced top decision makers, learning goal orientation is positively associated with ambidexterity, up to a point, after which it negatively effects ambidexterity. We argue this relationship exists because the benefits of learning goal orientation can eventually be outweighed by its costs. Too much risk and too much experimentation lead the firm to encounter more failure, and too much new information can be difficult to process, which makes it more difficult for a firm to integrate information that is critical to balancing exploration and exploitation activities. At the same time, we find that, for less experienced top decision makers, performance goal orientation is negatively related to ambidexterity, up to a point, after which it positively effects ambidexterity. We argue that this relationship exists because performance goal orientation is associated with a reluctance to take risks, lest a person demonstrate poor ability, and a reliance on existing capabilities. Therefore, increasing levels of performance goal orientation will reduce the overall willingness to innovate. However, at the highest levels of performance goal orientation, top decision makers will be compelled by an external orientation and an intense desire to compete, which will stimulate them to action, despite the possible risks. We also argue that top decision makers' role experience will flatten both relationships, as experience improves their decision making abilities and metacognitive capacity, mitigating the downsides of their goal orientations.

This study contributes to our understanding of ambidexterity by focusing on a highly relevant characteristic of top decision makers, their goal orientations. Just as ambidexterity concerns the simultaneous and complimentary activities of exploring for new opportunities while refining and strengthening existing exploitative activities, goal orientations research has explored the same questions at the individual level (Grant and Dweck, 2003). One reason ambidexterity – and strategic entrepreneurship, more broadly – is useful to firms is that both exploration and exploitation activities strengthen the other. Exploration provides the raw informational and experimental fuel that firms use to secure competitive advantages on the long run via exploitation, while exploitation provides the resources firms need to continually explore for new markets and new innovations (e.g., Taylor and Helfat, 2009; Hess and Rothaermel, 2011). Similarly, research on goal orientations has suggested that learning goal and performance goal orientation may function in the same way: that learning goal orientation can be useful in developing competencies, whereas performance goal orientation is useful in putting those competencies to effect in a competitive setting (Barron and Jarackiewicz, 2001). Therefore, goal orientations research may prove to be a fruitful and useful lens through which to further examine firm ambidexterity.

We also contribute to goal orientations research. Although management and organizational researchers have commonly found that many of the constructs they study may have adverse effects at extreme levels (or adverse effects become positive at extreme levels) (Pierce and Aguinis, 2013), a common assumption in goal orientations research is that the effects of learning goal and performance goal orientations are linear (e.g., Harackiewicz et al., 2002; Payne et al., 2007). Nevertheless, we offer hypotheses that argue top decision makers' goal orientations have non-linear effects on firm ambidexterity. Top decision makers, when motivated by very high levels of learning goal orientation, may lead their firms to excessive failure rates and hampering their firms' ability to process information, and our findings support this reasoning, at least among less-experienced top decision makers. Our findings also support the hypothesis that performance goal orientation, at very high levels, may be an antecedent of greater firm ambidexterity, especially among less-experienced top decision makers, as their desire to compete may overcome their hesitancy to take risks. The implications of non-linear goal orientation effects may provide an important update for the goal orientations literature, which has not deeply explored the possibility that goal orientations' effects may be non-linear.

Finally, the paper advances upper echelons research, especially research on top decision maker experience and tenure. In general, evidence has suggested that top decision makers with greater experience leading their firms may have little motivation to pursue innovative products or initiate strategic changes (Miller, 1991; Wang et al., 2016). Our findings help shed further light on the effects that top decision makers' experience can have on firm behaviors, and in some ways, our results are consistent with existing research. For example, while role experience mitigates the downside of top decision makers' performance goal orientation, it also mitigates the possible upsides as well. In other words, for experienced top decision makers, the motivation to prove themselves tends to affect firm-level outcomes less than it does for less experienced top decision makers. Alternatively, experienced top decision makers with high learning goal orientation tends to enhance firms' ambidexterity while mitigating any possible downsides. We drew partly on metacognition research (Haynie et al., 2010) to explain how experience affects these relationships, and the development of greater metacognition for experienced top decision makers may have many other effects, which we encourage future research to explore.

One practical implication of our study is that, while researchers have recommended firms emphasize hiring people high on learning goal orientation and avoiding people high on performance goal orientation (e.g., VandeWalle et al., 1999), these dynamics may be more complex and, according to our study, dependent upon people's role experience. While a firm interested in developing or maintaining an ambidextrous capability may consider hiring a executives who have high learning goal orientations, our results suggest these firms would be disappointed if they hired such an executive, but who had little role experience. Alternatively, inexperienced executives who are very high on performance goal orientation may help firms achieve ambidexterity, while more experienced executives may not.

Although the study is characterized by several strengths (multi-phase surveys spread across time, the sample of top decision makers), there are limitations. First, due to the nature of the study, we were unable to extensively explore the discrete mechanisms that link top decision makers' goal orientations and firm-level outcomes. While upper echelons theory provides a set of useful assumptions and prior research has elucidated these mechanisms, a greater focus on how goal orientations affect top decision makers' leadership and executive behaviors may be warranted. Relatedly, because our individual-level and firm-level variables were measured using the responses from firms' top decision makers, subsequent research may undertake multilevel methodological approaches in order to mitigate the possibility that goal orientations may influence how top decision makers assess their firms' ambidexterity. Second, due to the cross-sectional nature of our data, we are unable to assess changes between top decision makers' goal orientations and firm ambidexterity over time, which may be especially important, given our focus on goal orientations non-linear effects. Further research is needed to understand how, specifically, goal orientations influence people's behavior at increasingly higher degrees. For instance, as performance goal orientation increases, at what point might top decision makers become aware of the risks related to failure relative to the desire to best their competition? Third, we collected data from the same source – the top decision maker – concerning goal orientations and firm ambidexterity. This is a limitation because goal orientations can affect people's perception of their own behaviors, the risks they are taking, and their performance outcomes (cf. Lochbaum and Roberts, 1993). Fourth, firms hire top decision makers, and these hiring decisions can be influenced by a range of factors, including their preferences concerning innovation (e.g., Cummings and Knott, 2018). For instance, we find in our sample that older firms tend to have top decision makers with relatively lower learning goal orientation (r = −0.27 in Table 3) and performance goal orientation (r = −0.14 in Table 3), which could be an outcome of inertia and complexity that can exist in firms that have been long established (Voss and Voss, 2013). In other words, the firms in our sample could be hiring top decision makers that fit an existing innovative strategy rather than letting top decision makers shape innovative outputs, as we describe. Subsequent research could examine the interplay between firms' innovative activities and the cognitive and other personality attributes of the top executives they hire.

Despite these limitations, we hope this study contributes to scholars' understanding of the relationship between top decision makers' unique motivational attributes and firm ambidexterity. Ambidexterity is an important element of strategic entrepreneurship and firms' pursuit of value, and, although research has generally overlooked the top decision maker's role in fostering ambidexterity, this study highlights their role. Top decision makers are the nexus of information and decision making in their firm, and the ability a firm has in balancing exploration and exploitation activities is largely driven by their hand.

## Data Availability Statement

The raw data supporting the conclusions of this article will be made available by the authors, without undue reservation.

## Author Contributions

CP collected the data and wrote the first draft of the manuscript. SS and JX helped prepare the manuscript for submission. All authors contributed to the article and approved the submitted version.

## Conflict of Interest

The authors declare that the research was conducted in the absence of any commercial or financial relationships that could be construed as a potential conflict of interest.
